# Reactive oxygen species, glutathione, and vitamin E concentrations in dogs with hemolytic or nonhemolytic anemia

**DOI:** 10.1111/jvim.15926

**Published:** 2020-10-13

**Authors:** Andrew D. Woolcock, Priscila B. S. Serpa, Andrea P. Santos, John A. Christian, George E. Moore

**Affiliations:** ^1^ Department of Veterinary Clinical Sciences Purdue University College of Veterinary Medicine West Lafayette Indiana 47907 USA; ^2^ Department of Comparative Pathobiology Purdue University College of Veterinary Medicine West Lafayette Indiana 47907 USA; ^3^ Department of Veterinary Administration Purdue University College of Veterinary Medicine West Lafayette Indiana 47907 USA

**Keywords:** antioxidants, free radical, hemolysis

## Abstract

**Background:**

Red blood cells (RBC) are uniquely susceptible to oxidative injury. Oxidative stress is both a cause for, and effect, of anemia in people but this has been minimally documented in dogs.

**Objective:**

To describe direct and indirect markers of oxidative stress in anemic dogs.

**Hypothesis:**

Anemic dogs will have oxidative stress when compared to healthy dogs.

**Animals:**

Forty‐seven dogs with anemia (10 with hemolytic anemia) and 70 healthy control dogs.

**Methods:**

Prospective, cross‐sectional study. Anemic dogs were identified from the patient population, and medical records were reviewed to classify the anemia as hemolytic or nonhemolytic. Flow cytometry was used to detect reactive oxygen species (ROS) in erythrocyte isolates. Reduced glutathione (GSH) concentrations were measured in both plasma and hemolysate samples, and vitamin E was measured in serum.

**Results:**

Anemic dogs (both hemolytic and nonhemolytic) had significantly lower median RBC hemolysate GSH concentrations (3.1 μM [0.4‐30.8]) when compared to healthy dogs (7.0 μM [0.5‐29.7]; *P* = .03). Dogs with hemolytic anemia had significantly higher median plasma GSH (7.6 μM [0.4‐17.8]) when compared to dogs with nonhemolytic anemia (1.6 μM [0.01‐7.1]; *P* = .04) and healthy dogs (2.8 μM [0.1‐29.9]; *P* < .0001). Reactive oxygen species were detectable in all samples, but there was no difference in ROS or vitamin E between groups.

**Conclusions and Clinical Importance:**

Oxidative stress is present in anemic dogs. Derangements in biomarkers of oxidative stress are different in dogs with hemolytic anemia and nonhemolytic anemia.

**Abbreviations**
AKI: acute kidney injury
CKD: chronic kidney disease
CV: coefficient of variation
DCF‐: dichlorofluorescein
DCFH‐DA: 2′,7′‐dichlorofluorescein diacetate
DMSO: dimethyl sulfoxide
DTNB: 5,5′‐dithio‐bis‐2‐(nitrobenzoic acid)
EDTA: ethylenediaminetetraacetic acid
GPx: glutathione peroxidase
GSH: reduced glutathione
GSSG: oxidized glutathione
H_2_O_2_: hydrogen peroxide
Hct: hematocrit
HPLC‐FLD: high performance liquid chromatography and fluorescence spectrometer
IMHA: immune‐mediated hemolytic anemia
MDA: maldionaldehyde
MFI: median fluorescence intensity
PCV: packed cell volume
RBC: red blood cell
ROS: reactive oxygen species
TNB: 5‐thio‐2‐nitrobenzoic acid

## 1 INTRODUCTION

Oxidative stress occurs when there is an imbalance of systemic antioxidant and prooxidant factors.^1^, ^2^ Erythrocytes are at particular risk for oxidative injury because of their ubiquity, proximity to oxygen, lack of nuclear material, and high iron content.^1^, ^3^ Erythrocytes have antioxidant systems to prevent oxidative injury, the most important being the glutathione pathway.^4^ Glutathione is a tripeptide produced from cysteine, glycine, and glutamate, and is primarily formed and stored by the liver, but the red blood cells have intracellular glutathione as a major antioxidant defense.^5^ Glutathione exerts its antioxidant effect through neutralizing reactive oxygen species (ROS).^5^ Glutathione, in the presence of ROS is oxidized and the interaction of free radicals and enzymes like glutathione peroxidase form oxidized glutathione (GSSG). Oxidized glutathione can be recycled through the function of enzymes (eg, glutathione reductase) and cofactors (eg, vitamin C, vitamin E, and selenium), to be reduced to its original form (reduced glutathione, GSH).^2^, ^5^

When the accumulation of ROS exceeds antioxidant defenses, or when defenses are depleted, the erythrocyte will incur structural damage, typically through the oxidization of hemoglobin or peroxidation of the phospholipid membrane.^1^, ^4^ These changes reduce oxygen‐carrying capacity and can induce hemolysis or early senescence and apoptosis.^6^, ^7^ In dogs, oxidative stress is associated with several disease states, including diabetes mellitus, chronic kidney disease, congestive heart failure, neurodegenerative disorders, and hemolytic anemia.^8^, ^9^, ^10^, ^11^, ^12^, ^13^, ^14^, ^15^ In addition, oxidative stress can to contribute to the chronic anemia noted in several of the above diseases.^16^ Clinically, oxidative damage to the erythrocyte is recognized through red blood cell morphologic changes (eg, Heinz bodies and eccentrocytes), but markers of red blood cell or systemic oxidative stress in anemic dogs are minimally described.

In people, oxidative stress is described to be both a cause for and effect of anemia.^17^, ^18^ In anemia, there is an increase in oxygen demand by peripheral tissues.^19^, ^20^ This increased demand leads to vascular changes, namely vasodilation and increased production of nitric oxide. This increase in nitric oxide leads to an increased production of prooxidant compounds (ie, nitric oxide radicals and other ROS) and subsequent depletion of antioxidants.^19^, ^20^, ^21^ Oxidative stress to erythrocytes leads to the externalization of phosphatidylserine receptors, which signal for apoptosis.^7^ In addition, chronic structural injury to the erythrocyte membrane leads to antigenic epitope formation and autoantibody formation.^22^ Lastly, during hemolysis, free iron is released, which is a major substrate for ROS formation.^23^, ^24^, ^25^ Oxidative stress in anemic people is documented through direct measurement of erythrocytic ROS, and through depletion of the glutathione, antioxidant enzymes (including glutathione peroxidase), and increased byproducts of lipid peroxidation (eg, malondialdehyde [MDA], isoprostanes), and improves with antioxidant supplementation.^17^, ^22^, ^26^ In dogs, MDA increases in immune‐mediated hemolytic anemia, and glutathione peroxidase (GPx) activity decreases in a population of dogs with anemia by any cause.^12^, ^13^ GPx has a strong correlation with hematocrit but is measured on whole blood, raising concern that the decrease in GPx is a reflection of reduced red blood cell mass, rather than a real deficiency in this enzyme.^12^ There are no current studies that have evaluated a direct assessment of ROS, or that have evaluated biomarkers of oxidative stress in anemia in cellular components rather than whole blood. Antioxidant supplementation has not been evaluated in anemic dogs.

The purpose of this study was to evaluate oxidative stress, both directly and indirectly, in anemic dogs. We hypothesized that anemic dogs would have evidence of oxidative stress (increased intraerythrocytic ROS, decreased GSH, and decreased vitamin E) when compared to healthy dogs. We further hypothesized that dogs with hemolytic anemia would have alterations in these biomarkers of oxidative stress that differ from those in dogs with nonhemolytic anemia.

## 2 MATERIALS AND METHODS

### 2.1 Study design and populations

All skeletally mature dogs presenting to the Purdue University Veterinary Teaching Hospital (Purdue University, West Lafayette, Indiana) were eligible for the study. Dogs were included in the study if they were anemic which was defined as a packed cell volume (PCV) or hematocrit (HCT) of less than 30%. Dogs were excluded from the study if they were younger than 1 year of age or weighed less than 4 kg. Dogs were excluded if they had received a transfusion of any blood product within 7 days of presentation or if they had received any systemic corticosteroid within 3 days of presentation, or any long‐acting corticosteroid within 1 month of presentation. Other medications that prompted exclusion from the study included adjunctive immune‐suppressive medications, chemotherapeutic agents, and supplemental antioxidants (including Denamarin (Nutramax Laboratories, Lancaster, South Carolina) or Denosyl (Nutramax Laboratories, Lancaster, South Carolina), SAM‐e, omega 3 fatty acids, and vitamin E).

All anemic dogs had a complete blood count and a serum biochemistry profile performed on the day of the presentation. CBC and biochemistry profiles were performed by medical technologists on commercial analyzers (ADVIA 2020i, Siemens Medical Solutions USA, Malvern, Pennsylvania; VITROS 5.1 FS Chemistry System Analyzer, Siemens Medical Solutions USA, Malvern, Pennsylvania). Blood smears were prepared from each sample and were evaluated by a clinical pathologist using a modified Wright stain. Study samples were collected 1 time, on the day the anemia was identified. Based on clinical examination, laboratory findings, and the results of the diagnostic investigation performed at the clinicians' discretion, anemic dogs were subdivided into 2 groups: “hemolytic anemia” and “anemia because of nonhemolytic causes.” Anemia was defined as hemolytic if at least 1 of the following concurrent findings was identified: spherocytosis, positive saline agglutination test, hemoglobinemia/hemoglobinuria, or hyperbilirubinemia with no evidence of hepatobiliary disease. If the criteria for hemolysis were not fulfilled, the anemia was defined as nonhemolytic. The dogs with nonhemolytic anemia had their medical record reviewed for the final diagnosis and suspected cause for anemia.

A control population of skeletally mature, clinically healthy dogs was recruited from the age‐matched to the study population ±1 year. Dogs were included if they met the age and weight criteria described above and if they were clinically healthy and had not received any of the medications listed previously. Health status in these dogs was determined through a complete physical exam and assessment of a packed cell volume and total solids by refractometer.

Client consent was obtained for clinically healthy and anemic dogs. In addition, the study protocol was reviewed, approved, and conducted in accordance with the Purdue University Animal Care and Use Committee (PACUC Coeus Protocol Number 1509001296).

### 2.2 Sample collection and analysis

Blood samples were obtained via jugular venipuncture from all anemic dogs. Blood was collected into 2 EDTA tubes (Bectin, Dickinson and Company, Franklin Lakes, New Jersey) for ROS flow cytometry and GSH measurement, respectively, and into a serum tube (Bectin, Dickinson and Company, Franklin Lakes, New Jersey) for vitamin E measurement. One EDTA tube was centrifuged (3000*g* at 4°C for 5 minutes), and plasma was harvested and stored in cryotubes (Fisher Scientific, Pittsburgh, Pennsylvania) at −80°C until GSH analysis. From the remaining pellet of RBC, 200 μL was diluted 1:4 with 800 μL of ice‐cold HPLC‐grade water. After gentle rocking for 10 minutes, the dilution was centrifuged (10 000*g* at 4°C for 15 minutes). The supernatant, representing the hemolysate, was collected and stored in cryotubes at −80°C until GSH analysis. The second EDTA tube was used for flow cytometric measurement of ROS. The serum tube was allowed to sit for 20 minutes and then centrifuged (1000*g* at 4°C for 15 minutes), with serum harvested and stored in cryotubes at −80°C until vitamin E measurement. All samples were frozen within 1 hour of collection, and the vitamin E samples were shipped on dry ice. All samples were analyzed within 6 months of collection based on stability. The control dogs recruited for this study had blood collected by jugular venipuncture as described above, and blood was collected into 1 EDTA tube and 1 serum tube for gluthathione and vitamin E measurement. ROS flow cytometry of anemic dogs was compared to larger control population of 50 skeletally mature dogs (recruited using the same inclusion/exclusion criteria above) that were used to validate and establish expected normal values for the use of DCFH‐DA to identify intraerythrocytic ROS in dogs.

### 2.3 Intraerythrocytic reactive oxygen species measurement by flow cytometry

The EDTA tube was centrifuged (3000*g* at 4°C for 5 minutes), and the plasma and buffy coats were removed with a Pasteur pipette. Ten microliters of erythrocytes were diluted in 5 mL of phosphate buffered saline (PBS; Fisher Scientific, Pittsburgh, Pennsylvania) supplemented with 1% w/v bovine serum albumin (PBSA; Mallinckrodt Pharmaceuticals, St Louis, Missouri). All samples were allocated into 2 treatment groups characterized by either the presence of DCFH‐DA (Sigma‐Aldrich, St Louis, Missouri) or absence (DMSO; Millipore Sigma, Burlington, Massachusetts; vehicle control). Each sample was run in triplicate for each treatment.

Ten microliters of DCFH‐DA (25 μM) or DMSO were added to 5 mL round‐bottom tubes.

One hundred microliters of the diluted erythrocyte solution were then added to each tube. The cells were incubated at 37°C for 20 minutes. After incubation, the samples were quenched with 200 μL PBSA 1% and immediately analyzed by flow cytometry (BD Accuri C6 flow cytometer, Becton, Dickinson and Company, Franklin Lakes, New Jersey). Reactive oxygen species‐dependent fluorescence was detected by green fluorescence with an excitation wavelength of 488 nm with gating around erythrocytes only. The assay measures fluorescence induced by the interaction of the fluorochrome (DCFH‐DA) and any intracellular ROS, so it does not measure any specific reactive oxygen species, but rather the fluorescence correlates with the concentration of all ROS within the cells. All samples were analyzed within 2 hours of collection. The intraassay and interassay coefficient of variation (CV) are 11.8% and 11.8%, respectively (N = 50, data not provided).

### 2.4 Plasma and RBC hemolysate reduced glutathione concentrations

Reduced glutathione (GSH) was measured using a commercially available kit (Cayman Biochemical, Ann Arbor, Michigan), which utilizes a carefully optimized enzymatic recycling method, using glutathione reductase, for the quantification of GSH. The sulfhydryl group of GSH reacts with DTNB (5,5′‐dithio‐*bis*‐2‐(nitrobenzoic acid), Ellman's reagent) and produces a yellow colored 5‐thio‐2‐nitrobenzoic acid (TNB). The mixed disulfide, GSTNB (between GSH and TNB) that is concomitantly produced, is reduced by glutathione reductase to recycle the GSH and produce more TNB. The rate of TNB production is directly proportional to this recycling reaction, which is, in turn, directly proportional to the concentration of GSH in the sample. Because of the manipulation required to prepare plasma or hemolysates, it is not advised to also measure GSSG in these samples as manipulation of the sample can induce oxidation of glutathione postcollection. The absorbance of TNB was measured at 405 to 414 nm on a multiplate reader, and concentration determined from the formation of a standard curve. The interassay coefficiency of variation (CV) is 3.6% (N = 5) and intraassay CV is 1.6% (N = 84; Cayman Biochemical, Ann Arbor, Michigan).

### 2.5 Serum vitamin E concentrations

Vitamin E (as α‐tocopherol) was measured in serum by the Cornell University Animal Health and Diagnostic Center (Cornell University, Ithaca, New York).^27^ Briefly, Vitamin E concentrations were determined using high‐pressure liquid chromatography and fluorescence spectrometer (HPLC‐FLD). The analysis was performed in subdued light. Between 100 and 300 μL of serum was placed in a centrifuge tube, and 1 mL ethanol was added to the sample and vortexed for 10 seconds. Two milliliters of hexane was added and vortexed for 1 minute. Samples were placed in a rotating shaker for 5 minutes, then left undisturbed for layers to separate for 10 minutes. Hexane was removed by pipette, and then hexane extraction was repeated with 1 mL hexane. After shaking, samples were centrifuged at 2000 rpm for 5 minutes. Hexane removal was repeated, and the pooled hexane extracts were allowed to evaporate to dryness at 30 to 40°C under nitrogen. Six hundred microliters of a solution containing 684 mL acetonitrile, 220 mL tetrahydrofuran, 70 mL methanol, and 30 mL 1% w/v ammonium acetate solution were added to each tube and vortexed for approximately 1 minute. The resulting solution was transferred to an amber glass autosampler vial and capped for injection into the HPLC system. Aliquots of 30 μL of each working standard solution and each sample solution were injected. The peak area of the eluted peak for alpha‐tocopherol for all standard solutions and sample solutions was recorded.

### 2.6 Statistical analysis

Statistical power analysis was performed for sample size estimation. Extrapolating from previous studies investigating the glutathione pathway in dogs, a sample size of 24 dogs was determined for each of the groups (anemic and control; *P* = .05, power 90%).^14^, ^28^ Data were analyzed by a commercial software (Cayman Biochemical, Ann Arbor, Michigan). Normality was assessed using the Shapiro‐Wilk test. Values of selected variables were compared between groups of dogs (hemolytic anemia, nonhemolytic anemic dogs, and healthy control dogs). The Kruskal‐Wallis nonparametric ANOVA by ranks was used to compare groups if the data was not normally distributed (all variables except vitamin E concentrations), and if groups significantly differed then pairwise comparisons were conducted with Dunn's post hoc test adjusted for multiple comparisons. The unpaired *t* test was used for comparison if the data were normally distributed (vitamin E concentrations). Spearman rank correlation coefficients were calculated (rho). A value of *P* < .05 was considered significant.

## 3 RESULTS

Forty‐seven dogs with anemia and 70 healthy control dogs were enrolled in the study (Table 1). Breeds that were represented more than once in the anemic dogs included: mixed breed dog (14), Newfoundland (3), Airedale (2), Boxer (2), Cocker Spaniel (2), Labrador Retriever (2), and Shetland Sheepdog (2). There were no breed trends noted between the dogs diagnosed with hemolytic vs nonhemolytic anemia. Breeds that were represented more than once in the control dogs included: mixed breed dog (19), Golden Retriever (6), Rhodesian Ridgeback (4), Australian Cattle Dog (3), Beagle (3), German Shorthair Pointer (3), Pembroke Welsh Corgi (3), Australian Shepherd (2), Goldendoodle (2), Jack Russell Terrier (2), Labrador Retriever (2), and Shih Tzu (2). Ten anemic dogs were diagnosed with hemolytic anemia, and all 10 were presumed to have nonassociative disease based on the absence of associated comorbidities yielded from their diagnostic evaluation. The other 37 anemic dogs were classified as nonhemolytic. They had causes of anemia that included: kidney disease (both AKI and CKD are represented in this group, n = 9), nonperitoneal blood loss (including rodenticide toxicity, epistaxis, parvovirus with gastrointestinal hemorrhage, and trauma, n = 8), chronic inflammatory disease (including chronic skin issues, metritis, and chronic hepatopathy, n = 7), neoplasia (including lymphoma, mast cell tumor, and soft tissue sarcoma, n = 5), hemoabdomen (all were because of splenic masses, histopathology not consistently available, n = 5), and open diagnosis (n = 3). Anemic dogs were confirmed to have significant alterations on the complete blood count when compared to control dogs (Table 2). Twenty‐six anemic dogs were classified as nonregenerative with less than 100 000 reticulocytes/μL, while 19 anemic dogs had a regenerative anemia (median 228.9 × 10^3^/μL [112‐518.8]). Reticulocytes could not be determined in 2 cases because of agglutination. Red blood cell morphologic changes indicative of oxidative stress, including Heinz bodies and eccentrocytes, were noted infrequently on blood smear analysis (0 and 5 times, respectively) in anemic dogs, and none were noted on blood smear analysis of control dogs.

**TABLE 1:** Demographic information for the anemic (hemolytic and nonhemolytic) and healthy dogs is depicted

	Age (y)	Sex
All anemic (N = 47)	6.0 [1.0‐15.0]	22 F (5 intact) 25 M (2 intact)
Hemolytic anemia (N = 10)	7.7 ± 4.1^a^	4 F (1 intact) 6 M (1 intact)
Nonhemolytic anemia (N = 37)	4.6 ± 3.1^a,b^	18 F (4 intact) 19 M (1 intact)
Control (N = 70)	6.0^b^ [0.9‐14.0]	7 F (1 intact) 13 M (4 intact)

*Note*: Significant differences are denoted by matching superscripts. Age was not normally distributed when considered as anemic vs control (presented as median [range]), but individual anemic populations were normally distributed (presented as mean ± SD).

**TABLE 2:** Red blood cell (RBC) variables including RBC concentration, hematocrit (Hct), hemoglobin (Hgb), mean cell volume (MCV), and mean cell hemoglobin concentration (MCHC), as well as reticulocytes (Retic) are depicted as median [range] for the anemic and healthy dogs

	RBC (×10^6^/μL)	Hct (%)	Hgb (g/dL)	MCV (fL)	MCHC (g/dL)	Retic (×10^3^/μL)
	RI: 5.5‐8.5	RI: 37‐55	RI: 12‐18	RI: 60‐75	RI: 32‐36	RI: <100
All anemic (N = 47)	3.9^a^ [1.1‐4.9]	23.8^a^ [9.7‐29.9]	8.3^a^ [2.7‐10.8]	66.8^a^ [56.2‐94.4]	32.2 [28.2‐31.7]	84.9^a^ [4.0‐518.8]
Hemolytic anemia (N = 10)	2.4^b^ [1.1‐4.6]	17.8^b^ [9.7‐29.5]	5.3^b^ [2.7‐9.9]	77.6^b,d^ [64.9‐94.4]^b^	31.9^a^ [28.2‐37.2]	278.7^b,d^ [123.3‐518.8]
Nonhemolytic anemia (N = 37)	3.9^c^ [1.5‐4.9]	25.8^c^ [11.9‐29.9]	8.8^c^ [3.6‐10.8]	66.0^c,d^ [56.2‐78.8]^b^	33.6^a^ [29.3‐39.6]	59.5^c,d^ [4.0‐392.8]
Control (N = 70)	7.2^a,b,c^ [1.1‐9.6]	51.1^a,b,c^ [36.4‐64.8]	17.3^a,b,c^ [12.4‐21.6]	71.5^a,b,c^ [65.9‐77.1]	33.2 [31.7‐34.4]	45.1^a,b,c^ [9.6‐283.9]

*Note*: Anemic dogs had significantly lower RBC, hematocrit and hemoglobin, and significantly higher MCV and reticulocytes, compared to healthy dogs (*P* < .001). Dogs with hemolytic anemia had significantly higher MCV and reticulocytes, and lower MCHC compared to dogs with nonhemolytic anemia (*P* < .001). Matching superscripted letters denote comparisons that yielded statistical significance.

### 3.1 Biomarkers of oxidative stress

**FIGURE 1:**
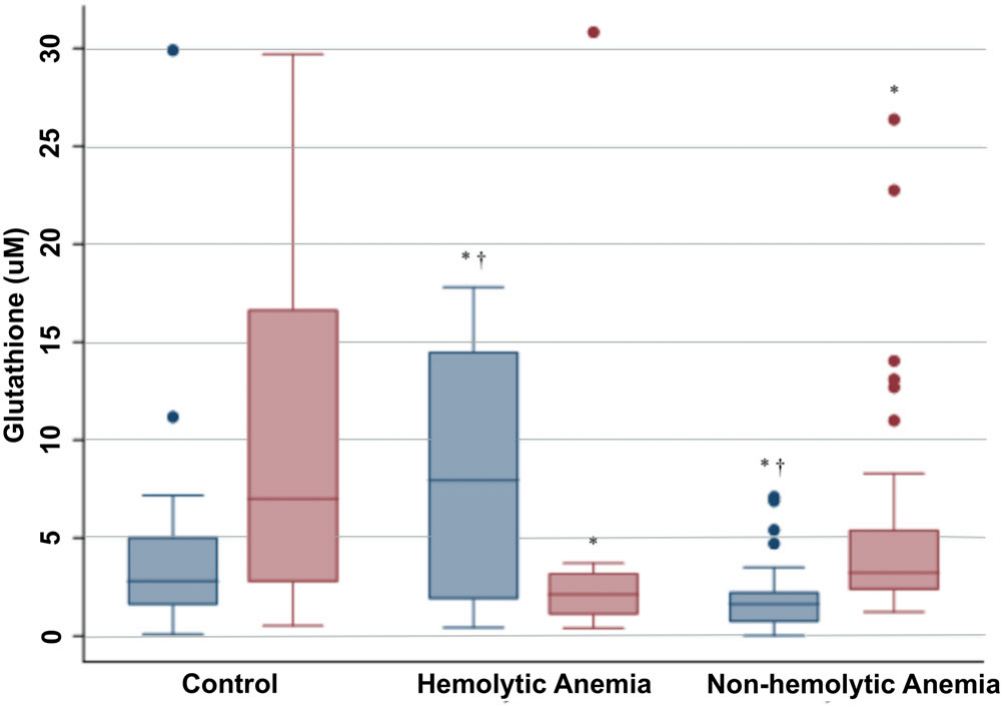
Plasma (blue boxes) and red blood cell (RBC) hemolysate (red boxes) reduced glutathione (GSH) concentrations for control dogs (N = 40), and dogs with hemolytic (N = 10) and nonhemolytic anemia (N = 37), respectively, are depicted in box‐and‐whisker plots. The top and bottom of each box represent the first and third quartiles, respectively, and the midline represents the median. The whiskers represent the range, with outliers represented by individual data points. An “*” denotes a significant difference when compared to healthy controls, and a “†” denotes a significant difference between anemia subtypes

Anemic dogs (all‐cause) had significantly lower RBC hemolysate GSH concentrations when compared to healthy dogs (*P* = .03, Table 3). Dogs with hemolytic and nonhemolytic anemia had similar RBC hemolysate GSH concentrations (*P* = .05). Dogs with nonhemolytic anemia had significantly lower plasma GSH concentrations when compared to healthy dogs (*P* = .02). Dogs with hemolytic anemia had significantly higher plasma GSH concentrations when compared to healthy dogs (*P* = .04) and dogs with nonhemolytic anemia (*P* < .0001, Figure 1). Intraerythrocytic ROS, as detected by flow cytometry, was not different between groups (Table 4). Serum vitamin E concentrations were not different between groups (Table 4). RBC hemolysate GSH concentrations had a significant weak positive correlation with hematocrit (rho = .26, *P* = .03) and hemoglobin concentration (rho = .26, *P* = .04). There were no differences in any biomarkers of oxidative stress when comparing dogs with nonregenerative anemia to those dogs with regenerative anemia.

**TABLE 3:** Median reduced glutathione (GSH) values from plasma and red blood cell (RBC) hemolysates from anemic and control dogs, further divided into those with hemolytic and nonhemolytic anemia

	Plasma GSH (μM)		RBC hemolysate GSH (μM)	
	Median	Range	Median	Range
All anemic (n = 47)	1.9	0.01‐17.8	3.1^d^	0.4‐30.8
Hemolytic anemia (n = 10)	7.6^a,c^	0.4–17.8	2.2^e^	0.4‐30.8
Nonhemolytic anemia (n = 37)	1.6^a,b^	0.01–7.1	3.2^f^	1.2‐26.4
Control (n = 20)	2.8^b,c^	0.1–29.9	7.0^d,e,f^	0.5‐29.7

*Note*: Anemic dogs had significantly lower RBC hemolysate GSH concentrations when compared to healthy dogs (*P* = .03). Dogs with a nonhemolytic anemia had significantly lower plasma GSH, and dogs with hemolytic anemia had significantly higher plasma GSH when compared to healthy dogs (*P* = .02, *P* = .04, respectively). Dogs with hemolytic anemia had significantly higher plasma GSH when compared to dogs with nonhemolytic anemia (*P* < .001). Values with matching superscript letters represent significant differences.

**TABLE 4:** Median intraerythrocytic reactive oxygen species (ROS) values presented as median fluorescence intensity from anemic and control dogs, further divided into those with hemolytic and nonhemolytic anemia

	RBC ROS MFI		Serum vitamin E (mg/dL)	
	Median	Range	Mean	SD
All anemic (n = 47)	2.50	1.73‐4.18	2813	±1474
Hemolytic anemia (n = 10)	2.61	1.73‐3.21	2993	±1525
Nonhemolytic anemia (n = 37)	2.45	1.74‐4.18	2589	±1422
Control (n = 50)	2.94	2.66‐3.42	N/A	RI: 500‐2400

*Note*: Additionally, mean serum vitamin E concentrations are presented from anemic dogs, along with the laboratory reference interval for canine serum vitamin E concentrations. There were no significant differences between or within groups.

## 4 DISCUSSION

In this population of anemic dogs, RBC GSH was significantly decreased compared to that of healthy dogs. This finding is similar to the glutathione deficiency reported in people with anemia, and supports the previous finding of decreased GPx activity in anemic dogs, as GPx is required for the recycling of GSH from its oxidized to reduced forms.^12^, ^17^, ^22^ In the present study, equal volumes of RBC were lysed for measurement of RBC GSH, indicating this deficiency is not just an artificial reflection of reduced red blood cell mass. In addition, the weak positive correlation between RBC GSH and markers of red cell mass, including hematocrit and hemoglobin, supports the conclusion that oxidative stress increases as anemia worsens. In people, it is theorized that RBC are systemic modulators of oxidative stress.^16^ The intricate antioxidant systems within RBC help to balance their redox state, but can also provide antioxidant protection to other cells and intercellular systems of the body.^4^ RBC have this function because of their mobility, occurrence throughout the body, renewability, and their vast number.^22^ Therefore, anemic states and those in which erythropoiesis is either reduced, absent or impaired can greatly impact the overall oxidative stress of the body.

The dogs with hemolytic anemia had the lowest RBC GSH concentrations, but also significantly elevated concentrations of GSH in plasma. The accumulation of GSH in the plasma is likely because of the release of intracellular GSH during hemolysis as the red blood cell mass reduced over time.^5^, ^21^, ^29^ Plasma GSH is increased in people with hemolytic anemia, though GSH is more traditionally measured in whole blood.^21^ It was important for this study to evaluate separate fractions of GSH to understand the effect that red blood cell mass has on these values. The duration of hemolysis was unknown in these cases, so future studies should investigate the effect of acute vs chronic hemolysis on the plasma and RBC fractions of GSH. It is unclear whether or not the free plasma GSH can impart similar antioxidant function as when it is intracellular.^5^ Extracellular glutathione is typically at least 1 order of magnitude lower than intracellular glutathione in most mammalian species, and in the extracellular spaces, GSH is often cleaved, and the by‐products are recycled by the liver for new tripeptide synthesis.^5^

ROS were detected in erythrocytes in all cells, but the fluorescence intensity was not different between anemic and control dogs. The anemic dogs had a wider range of values identified, but medians were similar among groups. It is unclear as to why a difference was not identified. Additionally, measuring ROS in isolated RBC might remove a primary source of ROS in anemic and inflammatory disease states, as neutrophils contribute peroxidase which subject surrounding cells to oxidant risk. Additionally, a significant difference could not have been detected as we believe the methodology could be improved. In people, flow cytometry with DCFH‐DA has been used to measure ROS in anemia directly.^30^, ^31^ However, in healthy people, ROS could not be detected in healthy RBC without the addition of a stimulus of oxidative stress (typically H_2_O_2_).^31^ In these studies, a linear model was used to measure ROS MFI, and the flow cytometer was not as sensitive as the 1 used in our study.^31^ In our validation of this assay in healthy dogs (submitted for publication), cells were incubated first with DCFH‐DA, similar to the current study, but were then incubated at room temperature with H_2_O_2_ (0.5 mM). This stimulation reliably induced detectable ROS formation, with values much higher than what was detected in this study. It is possible that flow cytometry is unable to detect subtle increases in ROS, like what could happen in anemic dogs, and that a more potent stimulus like H_2_O_2_ is required for reliable detection. In people, flow cytometry is used to detect RBC ROS in disease states, and H_2_O_2_ is used to stimulate oxidation, even if an endogenous source of oxidative stress is suspected. This stimulation introduces an oxidative challenge as moreso a measure of the cellular antioxidant capacity, rather than a true measurement of ROS.^31^ Therefore, we believe that this assay should be repeated in anemic dogs with H_2_O_2_ used as an oxidant challenge, and use this response as a marker of the cellular redox balance.

Serum vitamin E concentrations were not decreased in anemic dogs as hypothesized. In fact, mean serum vitamin E concentrations were slightly above the expected reference interval in the anemic dogs. Vitamin E deficiency (often because of malnutrition) is a reported cause for hemolysis, and vitamin E is an important antioxidant and cofactor for proper GSH function.^13^, ^32^, ^33^ In people, vitamin E deficiency is well described as a cause for anemia but does not appear to be an expected sequela unless there is a concurrent hepatobiliary disease.^32^, ^33^ In a previous study of dogs with IMHA, vitamin E levels were normal despite evidence of increased lipid peroxidation.^13^ It would appear that vitamin E concentrations are not a useful biomarker in anemic dogs unless it is believed that a deficiency in this vitamin could be a contributor or cause.

This study had several limitations. The sample size was adequate to detect differences between anemic and healthy dogs, but the subgroup analysis only included 10 dogs with hemolytic anemia. Future studies should include a higher number of dogs with hemolysis to gain a better understanding of the intracellular and extracellular redox changes that occur in a state of hemolysis. Additionally, a group of nonanemic, systemically ill patients with similar comorbid diseases could be investigated to further investigate other factors like fever, inflammation, iron deficiency, and so forth which could contribute to oxidative stress. Additionally, the use of flow cytometry and DCFH‐DA for detection of intraerythrocytic ROS has recently been validated in dogs, and this represents an initial foray into the use of the assay in canine disease states. Because of this, the results were inconclusive in anemic dogs, so further testing and adjustments to methodology will be required to understand the utility of this assay in disease states.

In conclusion, reduced glutathione (GSH) concentrations are decreased in anemic dogs, more so in hemolytic states. This likely represents antioxidant depletion and oxidative stress in anemia. Future studies should focus on optimizing the direct assessment of ROS. Antioxidant supplementation could be considered to determine the role that oxidative stress has in contributing to anemia in dogs.

## CONFLICT OF INTEREST DECLARATION

George Moore serves as Consulting Editor for Experimental Design and Statistics for the Journal of Veterinary Internal Medicine. He was not involved in review of this manuscript.

## OFF‐LABEL ANTIMICROBIAL USE DECLARATION

The authors declare no use of off‐label antimicrobials.

## INSTITUTIONAL ANIMAL CARE AND USE COMMITTEE (IACUC) OR OTHER APPROVAL DECLARATION

Protocol 1 509 001 296 approved by Purdue University College of Veterinary Medicine.

## HUMAN ETHICS APPROVAL DECLARATION

Authors declare human ethics approval was not needed for this study.
